# Lung recruitment mechanics: coalescing tissue strains with organ expansion

**DOI:** 10.1186/s12931-025-03118-8

**Published:** 2025-02-18

**Authors:** M. Shankel, T. M. Nelson, K. A. M. Quiros, J. Bebawy, C. A. Mariano, T. Biddle, D. D. Lo, M. Eskandari

**Affiliations:** 1 Department of Mechanical Engineering, University of California, 900 University Ave, Riverside, CA 92506 USA; 2 Breathe Center, School of Medicine, University of California, Riverside, CA USA; 3 Department of Bioengineering, University of California, Riverside, CA USA

**Keywords:** Mechanical ventilation, Ventilator induced lung injury, Pressure-volume response, Stepwise recruitment, Overdistension

## Abstract

**Background:**

Recruitment maneuvers are used to prevent atelectasis, or partial lung collapse, and to help prevent ventilator induced lung injury. Recruitment techniques remain a topic of debate due to the possibility for damage as they necessitate higher transpulmonary pressures, which are associated with inducing lung injury. We aim to evaluate and probe injury mechanisms and potential pressure inhomogeneities, expressed as heterogeneous lung recruitment and overdistension, by associating organ level compliances with continuous regional strains during the application of stepwise escalation contrasted with sustained inflation maneuvers.

**Methods:**

An established breathing mimicry electromechanical system integrated with high spatio-temporal digital image correlation techniques coupled the global pressure-volume response of the lung with local deformations. Compliances, pressures, strains, heterogeneities and the expansion evolution pertaining to the inflation phase of two recruitment methods were quantified and contrasted.

**Results:**

Significant differences between the organ- and tissue-level responses of the sustained inflation versus escalation maneuver were found. The escalation maneuver exhibited greater starting compliance, whereas the sustained inflation showed increased inflation compliance. The localized strain distribution for the sustained inflation yielded increased 75th percentile strain, 90th percentile strain, and range at maximum inflation compared to the escalation maneuver.

**Conclusions:**

Local and global findings indicate the escalation maneuver exhibits more homogeneous lung recruitment compared to sustained inflation. We also observe a correspondence between the significant organ-level compliance differences between the two maneuvers and the disparities observed in the evolutionary progression of localized strain distributions throughout inflation.

## Introduction

Lung diseases are a leading cause of death in the United States, where severe forms may necessitate mechanical ventilation (MV), emphasizing the need for research on optimal MV settings to ensure patient safety and facilitate recovery [1–2]. Previous research has led to employing lung protective ventilation strategies, which can consist of low tidal volumes and increased positive-end expiratory pressure (PEEP). These strategies reduce ventilator induced lung injury (VILI) occurrences and help keep the lung open [3]. Although this may be considered a safe technique, the usage of reduced tidal volumes can lead to atelectasis, or partial lung collapse, which can cause ineffective MV and damage through alveolar overdistension (heightened stress and strain) and atelectrauma (cyclic opening and closing of alveoli) [4–7]. Therefore, recruitment maneuvers are utilized to re-open collapsed alveolar groups and avoid atelectasis [8].

Currently, two commonly used recruitment maneuvers are the escalation recruitment maneuver (EM) and the sustained inflation recruitment maneuver (SIM). EM involves stepwise increases, usually in PEEP, to recruit alveoli, while SIM entails a volumetric hold to fully recruit the lungs [9–11]. However, the optimal method for recruiting the collapsed areas of the lung remains an ongoing debate and a challenge, including the paradoxical occurrence of unintended lung injury when implementing such maneuvers.

EM has demonstrated general advantages in promoting safer recruitment despite requiring an extended time implementation duration and manual implementation [10, 12]. Alternatively, SIM is included as a setting on many ventilators, increasing accessibility [13]; however, recent studies call for its discontinuation. SIM is no longer recommended according to acute respiratory distress syndrome (ARDS) guidelines, theorized that its usage is associated with lung damage leading to complications and prolonged post-operative recovery periods for patients [11–14].

Previous investigations sought to characterize recruitment maneuvers and their efficacy through multiple modals of measurement. Cereda et al. utilized magnetic resonance imaging (MRI) during an applied EM to demonstrate air distribution into all parts of the lung, inferring EM managed to open closed smaller alveolar groups as opposed to over distending previously opened groups [15]. Rimensberger et al. utilized global pressure-volume curves to assess lung mechanics following SIM; they found SIM provided subsequent protection from lung injury and enabled the usage of lower PEEP and tidal volume settings after recruitment [16]. However, these studies yield only snapshots instead of continuous assessments to understand how heterogeneity and overdistension occur and evolve, as can be yielded by continuous measurements of pulmonary surface stretch during inflation.

In this current study, we unify the relationship between localized lung tissue strains and global pressure-volume curves during the inflation phase of two methods of lung recruitment: EM and SIM. Our custom-designed electromechanical system gathers continuous global pressure-volume curves uniquely integrated with digital image correlation (DIC) to quantify continuous deformation at the tissue level as associated with the bulk organ response [17–18]. Quantifying this continuous regional deformation can offer insights regarding the viscoelastic features of the lung, where we hypothesize that SIM is associated with increased pressure inhomogeneities, expressed as more heterogenous recruitment and surface overdistension in comparison to EM.

## Methods

**Fig. 1:**
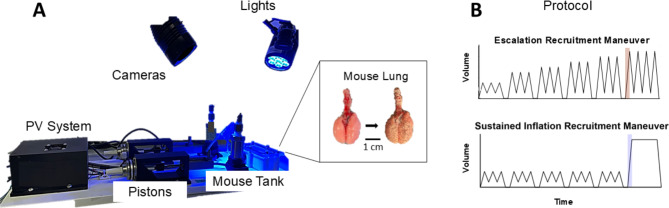
Experimental setup and testing protocol. (**A**) The electromechanical pressure-volume device is attached to the tank housing a speckled mouse lung. (**B**) The test protocol, whereby the lungs underwent either an escalation recruitment maneuver (EM) or a sustained inflation recruitment maneuver (SIM), is shown. The highlighted portion with analogous comparable states between EM and SIM protocols indicates the stage of analysis for global and local mechanics

C57BL/6J male mice, aged 12–15 weeks, were obtained from Jackson Laboratories (Bar Harbor, ME, USA) and sacrificed according to IACUC protocol (#20210011), where animal usage followed ARRIVE guidelines [19]. As in previous studies, extracted lungs were cannulated and inflated with 0.5 mL of air for speckling with waterproof paint for digital image correlation (DIC) measurements [17]. Lungs were hydrated in 1X phosphate buffered saline and ventilated with our custom-designed electromechanical system (Fig. 1A) [20].

Seven specimens were subjected to EM and another six underwent SIM, with separate groups used to ensure consistent pre-conditioning between lungs, and to avoid the influence of sequential recruitment maneuvers (Fig. 1B) [11, 21–23]. In accordance with previous studies, both maneuvers were performed at 20 breaths per minute (BPM), with preload (PEEP) of 6 cm H_2_O (24–25). Both EM and SIM applied 0.3mL preconditioning inflation-deflation cycles [25–28] for a shared datum state. Subsequently, EM specimens underwent 0.1mL stepwise cycle increases from 0.5 mL to 0.9 mL (11, 15, 24–25), whereas SIM specimens maintained 0.3mL cycles to match the EM group’s history before being inflated to the same 0.9mL abruptly [21–23]. The resulting analogously matched EM and SIM inflation phases were analyzed (Fig. 1B; red and blue highlighted stage).

Volume-time (VT), pressure-volume (PV), and pressure-time (PT) curves were collected from each ventilation scheme (where lung volume was directly measured with our airtight two-piston apparatus, accounting for air and tissue compressibility [20, 29]) and analyzed utilizing MATLAB R2023a (MATLAB, MathWorks Inc., Natick, MA, USA). A bilinear fit was applied to the PV curve to extract the starting compliance (*C*_*Start*_) and inflation compliance (*C*_*Inflation*_); the static compliance (*C*) was assessed as the ratio of the response volume to the peak transpulmonary pressure [30–33]. Peak pressures and R^2^ values denoting the degree of linearity of the PT curve (and associated with what is termed the stress index and homogenous recruitment) were evaluated [34–36].

Local tissue technical strains were recorded (20 Hz, 4096 × 3000 pixel resolution, Trilion Quality Systems, GOM ARAMIS 2016, King of Prussia, PA, USA); the reduced Jacobian was calculated to ensure equivalent comparisons between the surface areas and displaced volume for each specimen [29, 37]. Non-physiological strain outliers linked to surface component edge noise were discarded. Representative temporally evolving strains, discrete inflation stage histograms, and strain distribution plots were generated for each recruitment maneuver [18]. At maximum inflation, histograms of major and minor strains were calculated [25], where major and minor strains are defined as orthogonal maximum and minimum principal strains whereby the shear stress is zero, as defined in traditional mechanics (17–18). The mean, median, 75th percentile, 90th percentile, and range values were calculated and reported for major strain. Anisotropy serves as a measure of distortion and a potential disease marker in humans, and was calculated as the ratio of minor to major stretch instead of strain, to avoid negative anisotropic values; and histograms of the surface fraction versus anisotropic ratio were generated, where a value of one indicates isotropic behavior [27, 29, 37–38]. Associations between the local mean strain, along with 75th and 90th percentile strain values coupled to global pressure and volumes over the full inflation range were plotted.

Statistical significance was analyzed using a parametric unpaired t-test with significance thresholds defined at *p < 0.05 and **p < 0.01, and where normality was verified via the Shapiro-Wilk test [GraphPad Prism, San Diego, CA, USA] [39]. Further, to assess for significant correlation between global metrics and local overdistension, the Spearman’s correlation test compared the 90th percentile strain value with the R^2^ pressure-time curve linear fit, inflation compliance and starting compliance [40–41].

## Results

### EM versus SIM pressure-volume inflation patterns

**Fig. 2:**
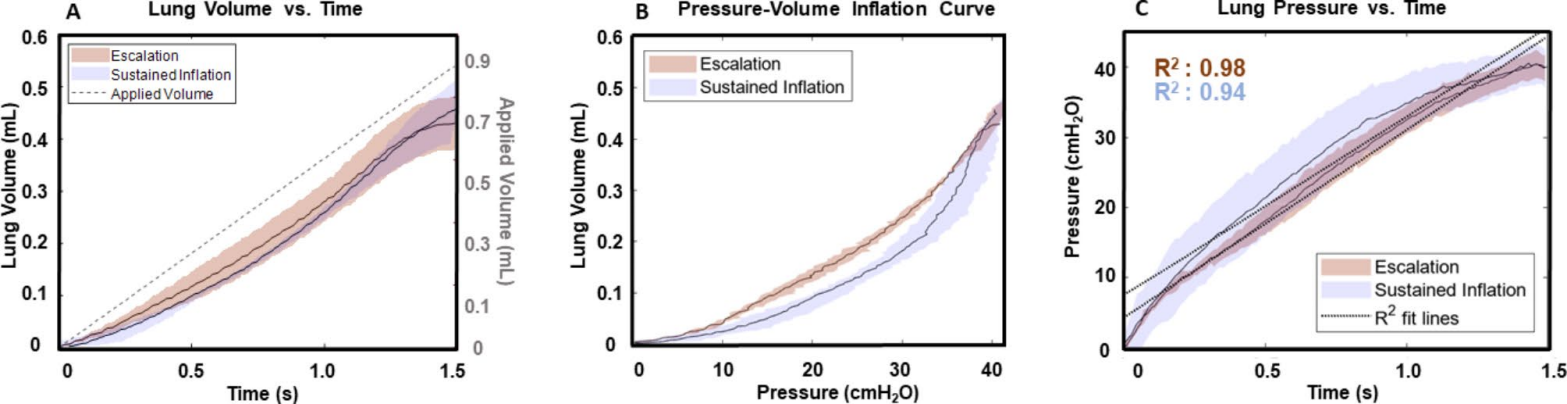
Average global lung responses recorded from the custom pressure-volume device (shaded +/- standard deviation) contrasting EM and SIM. (**A**) Measures of lung volume over the inflation duration; (**B**) Pressure versus lung volume curve for each maneuver during inflation; and (**C**) The temporal lung pressure for each maneuver

Figure 2 compares the applied volume and lung volume measured over time (VT), the lung volume versus pressure (PV), and the lung pressure evolution (PT) inflation measures between EM and SIM. EM increased lung volume more linearly and rapidly compared to the SIM response (Fig. 2A). EM and SIM demonstrated similar PV non-linear convex curves, albeit with differing starting and inflation compliances: SIM compliance slopes were initially shallower and conclude more steeply than EM curves (Fig. 2B). Concave PT curves (Fig. 2C) found EM was more linear with an R^2^ value of 0.98, compared to SIM where R^2^ was 0.94.

**Fig. 3:**
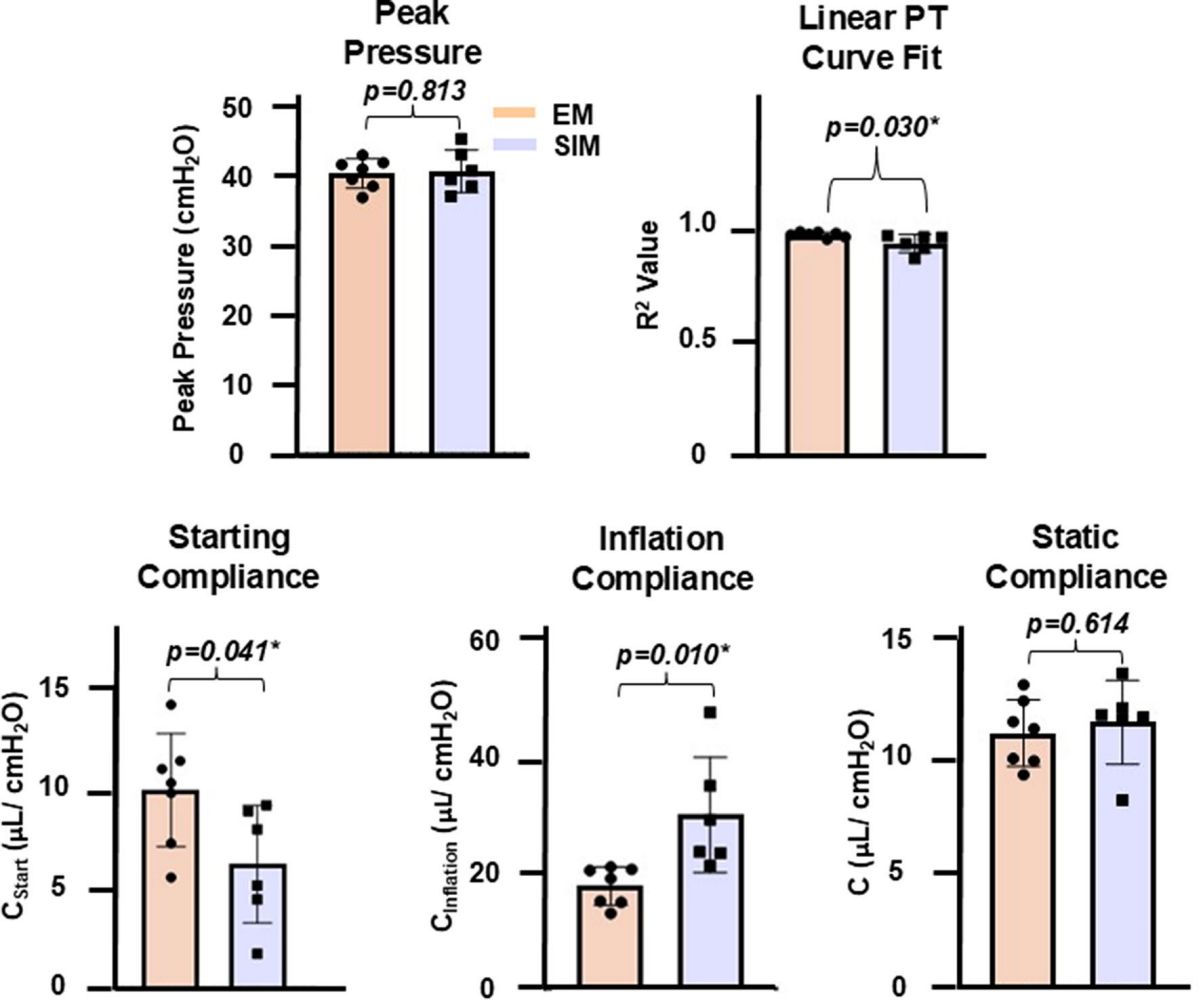
Reported values and significance for peak pressure, R^2^ linear PT curve fit, starting compliance, inflation compliance, and static compliance for both EM and SIM.

Differences between EM and SIM for starting compliance, inflation compliance, and the R^2^ value for a linear fit to the PT curve, were found to be statistically significant, with EM resulting in a higher starting compliance and R^2^ linear fit value, and SIM resulting in a higher inflation compliance (Fig. 3 p-values listed; Table 1). The peak pressure and static compliance differences between EM and SIM were found to not be statistically significant (Fig. 3), although SIM trended slightly higher average static compliance.

**Table 1:**
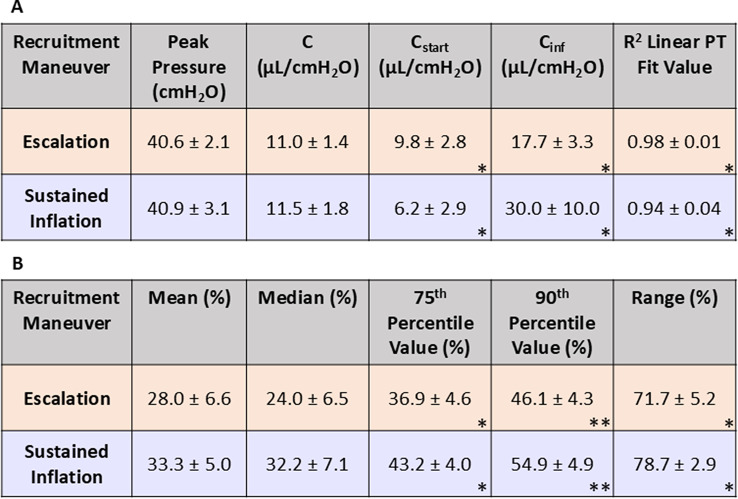
Resulting averaged values +/- standard deviation for global and local parameters, where * indicates *p* < 0.05 and ** indicates *p* < 0.01 differences between EM and SIM: (A) values for peak pressure, static compliance (*C*), starting compliance (*C*_*start*_), inflation compliance (*C*_*inf*_) and the R^2^ linear PT fit value; (B) values for major strain at maximum inflation for mean, median, 75th percentile, 90th percentile and the range

### Local strain temporal evolution and distributions

**Fig. 4:**
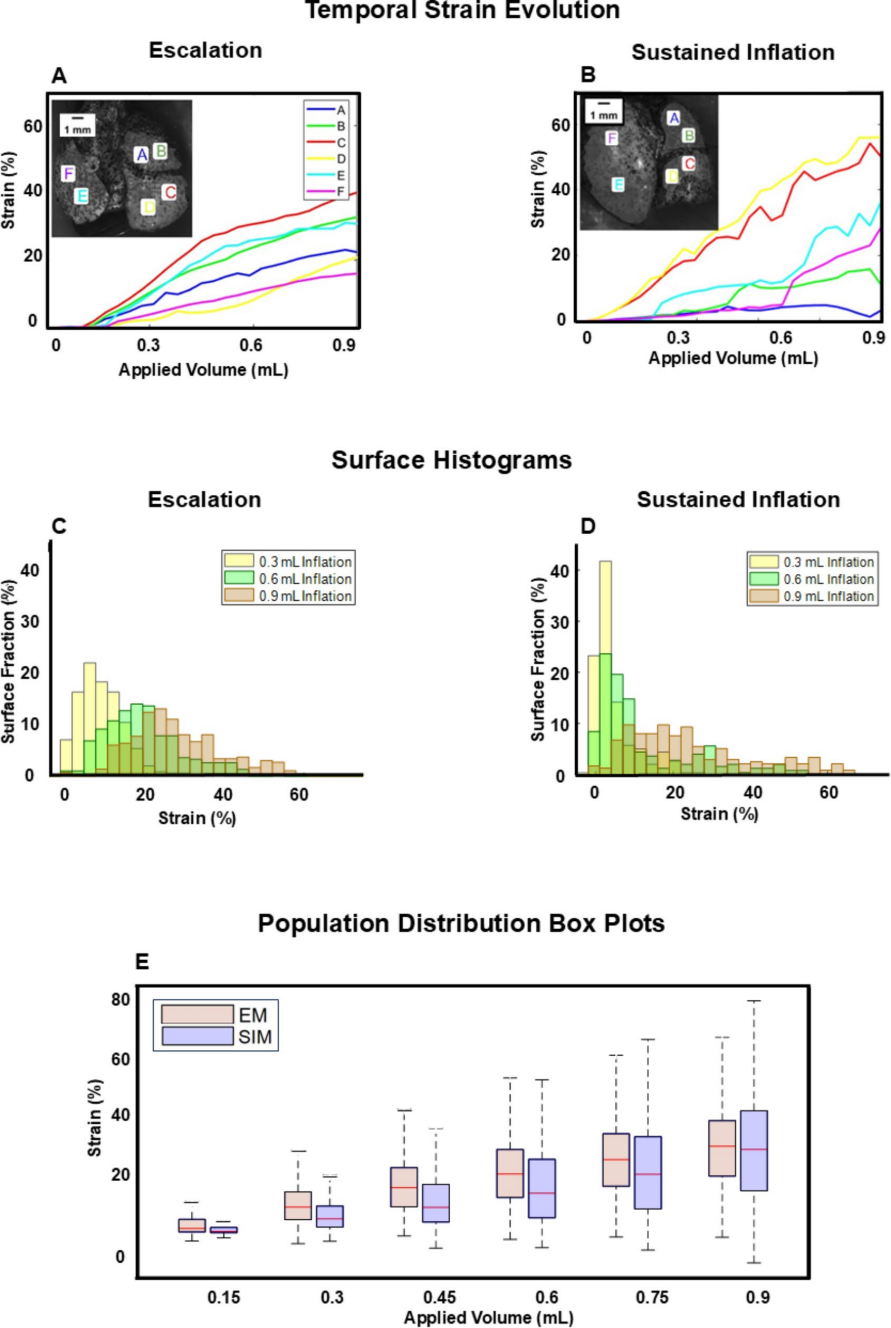
Evolving strain behavior of each recruitment maneuver: (**A**) and (**B**) Temporal strain evolution for representative specimens with select locations on the left lung and the superior and inferior right lobes observing steady EM strain increase compared to more discontinuous SIM. (**C**) and (**D**) Surface strain histograms of representative mice at increasing applied volumes stages contrast EM and SIM strain distributions. (**E**) Changing strain distributions throughout inflation across all specimens exhibit contrasting median, quartile values, and range trends for EM and SIM

Both ventilation maneuvers exhibited increasing local strains over time (Fig. 4A and B), in conjunction with increasing lung volume, as expected. EM selected strain locations were observed to exhibit a convex shape, with a slope decrease approximately midway through inflation. In comparison, SIM strains were observed to demonstrate a less uniform response, with a variance in slope patterns manifesting in greater heterogeneity at maximum inflation.

Representative histograms of the strain distribution as a fraction of the surface demonstrated a symmetric, unimodal distribution of strains for EM at discrete applied volumes of 0.3, 0.6 and 0.9 mL (Fig. 4C). In contrast, SIM exhibited a higher fraction of the lung surface at low strain values until 0.9 mL (maximum inflation), wherein SIM resulted in a wider strain distribution (Fig. 4D).

Population box plots of all samples revealed increasing median, quartile values, and range with increased applied volume for both maneuvers (Fig. 4E). EM resulted in a more uniform increase in all metrics, whereas SIM exhibited a greater increase in the 75th percentile metric compared to the 25th percentile as the applied volume increased, concluding in a greater interquartile range.

**Fig. 5:**
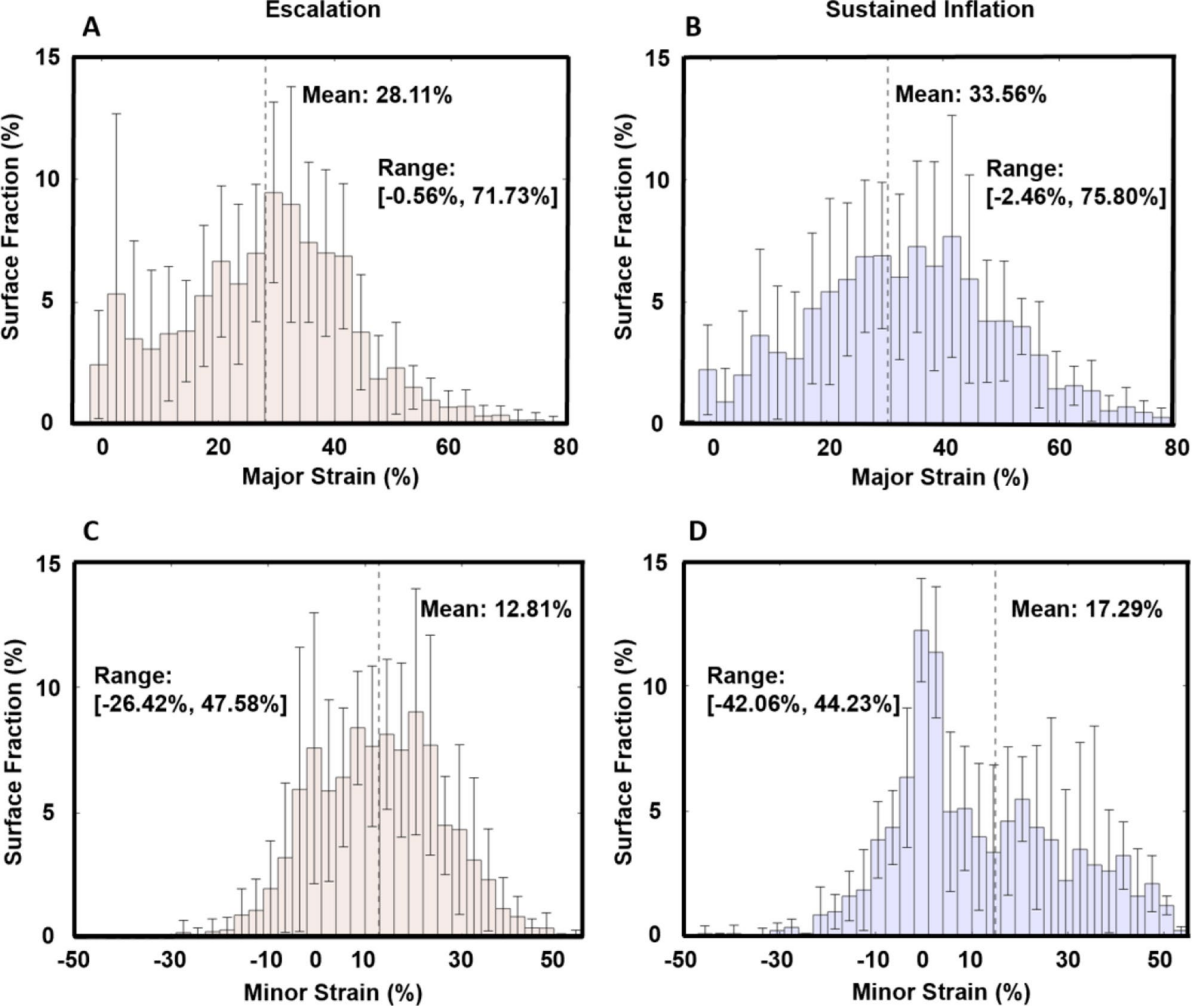
Histograms generated at maximum inflation (+/- standard deviation) for each maneuver: (**A**) and (**B**) Major strain distribution as a fraction of the surface; (**C**) and (**D**) Minor strain distribution as a fraction of the surface

At maximum inflation, the EM major strain histogram demonstrated peak surface fraction percentages distributed near the mean strain, with few surface values measuring above 40% major strain (Fig. 5A). In contrast, the SIM major strain histogram demonstrated a wider range of distributed values and an increased population in strain values above 40% (Fig. 5B). Minor strain histograms (Fig. 5C and D) of EM and SIM exhibited a similar trend to major strain histograms; however, while SIM maintained a greater range of values, there was a notable peak around 0% minor strain.

**Fig. 6:**
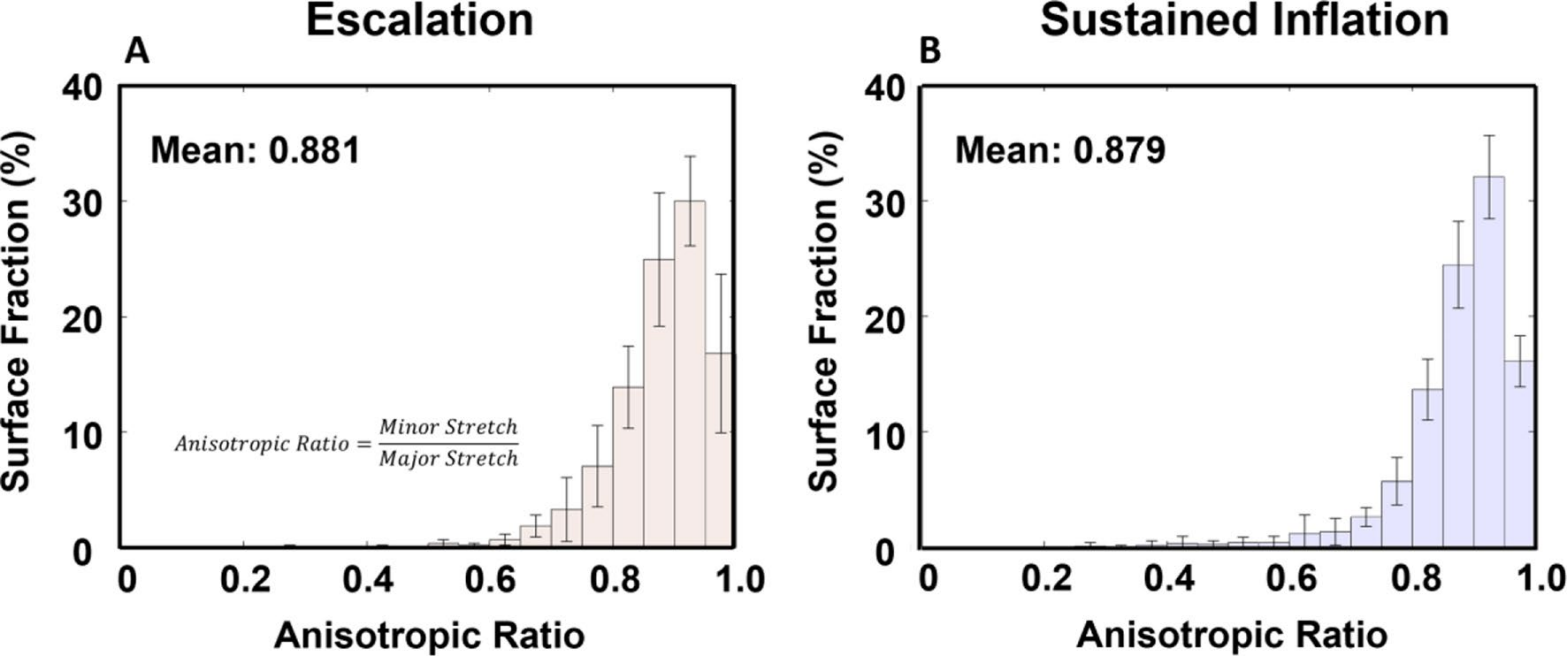
Strain anisotropy expressed as a histogram. (**A**) The escalation maneuver anisotropy. (**B**) The sustained inflation maneuver anisotropy

Figure 6 demonstrated strain anisotropy distribution with near identical trends for EM and SIM, with mean anisotropic ratio values of 0.881 and 0.879 for EM and SIM respectively.

### Local statistics and global-local plots

**Fig. 7:**
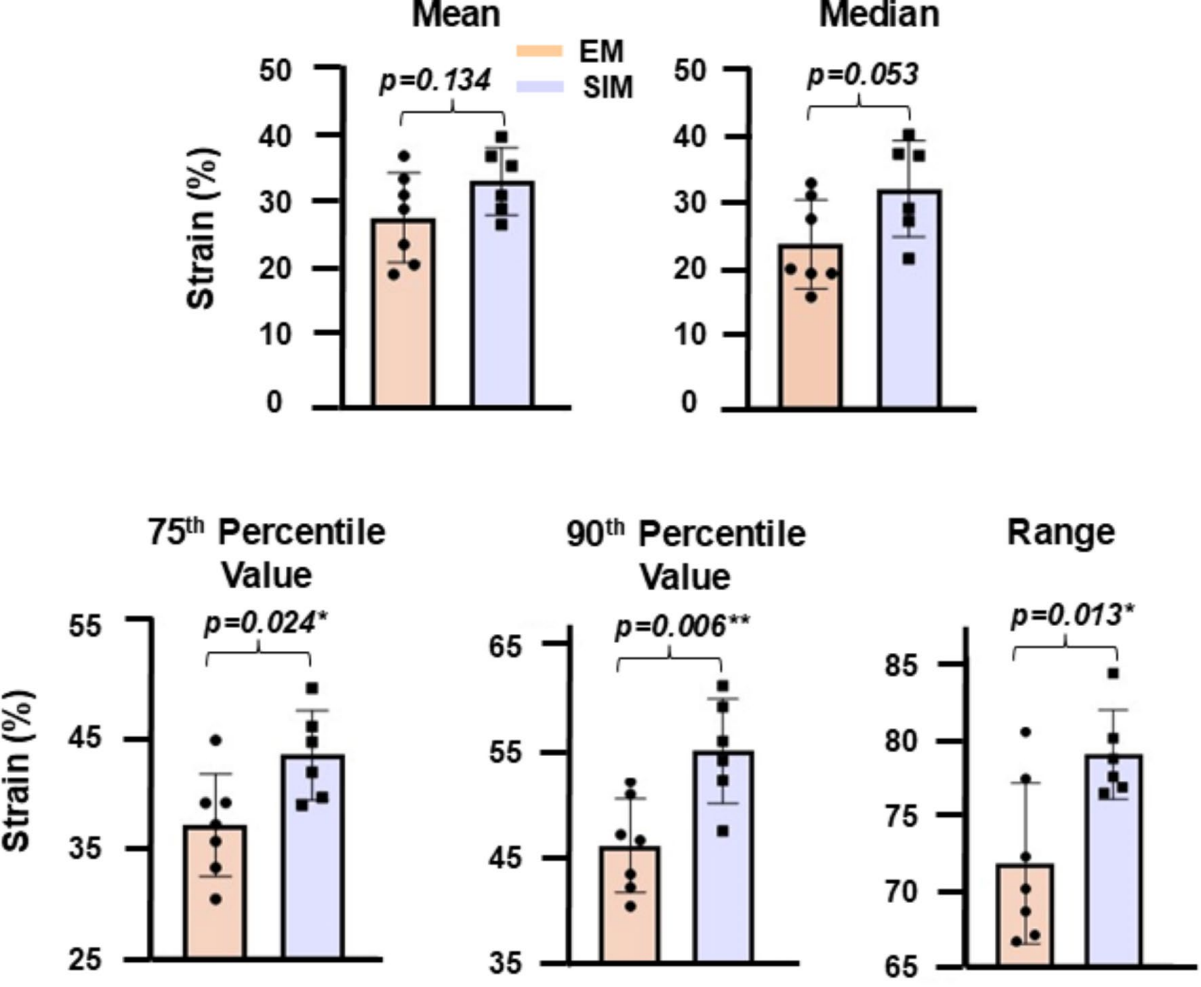
EM and SIM local major strain statistics generated at 0.9 mL applied volume (maximum inflation) for the mean, median, 75th percentile, 90th percentile, and the range

Statistically significant differences at maximum inflation were found for the 75th percentile, 90th percentile, and range strain values, where SIM exhibited greater values than EM. Statistical significance was not observed for the mean or median, meaning significantly greater population of peak strain values were seen for SIM, while at non-significantly different mean strain values (Fig. 7).

**Fig. 8:**
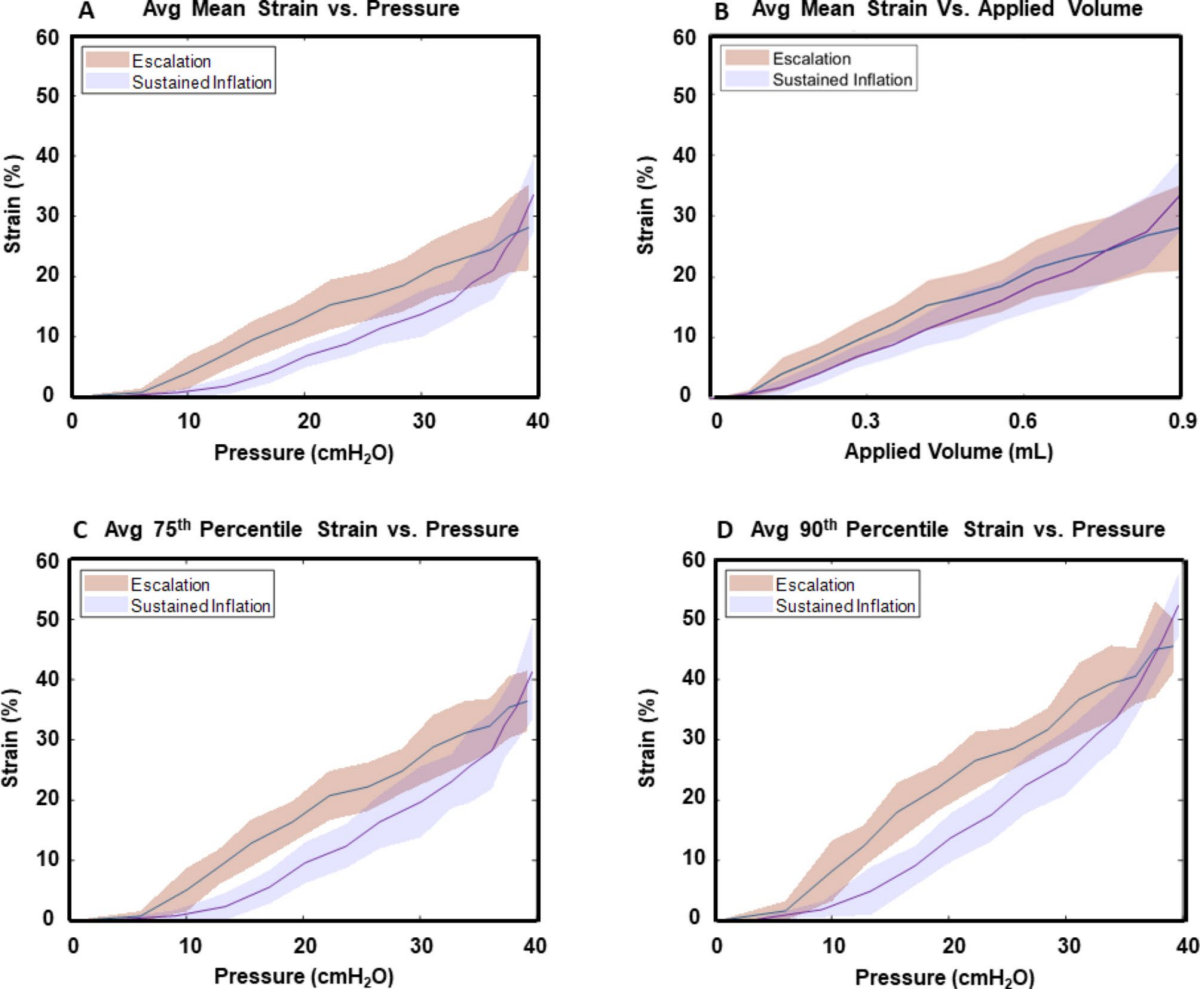
Associated global pressure-volume data with the localized strain data (average +/- standard deviation): (**A**) the mean strain versus the pressure; (**B**) the mean strain over the applied volume; (**C**) the 75th percentile major strain value as the pressure evolves; (**D**) the 90th percentile major strain value as a function of pressure

The mean strain increased with increasing pressure and time and followed similar trends as the PV curve and VT curve (Fig. 8A and B). The 75th percentile and 90th percentile demonstrated similar trends, but had qualitatively sharper increases in slope for SIM at high pressures, resulting in significantly greater peak values (Fig. 8C and D). After initial inflation, SIM generally appears more convex, particularly at maximum inflation, compared to EM.

**Fig. 9:**
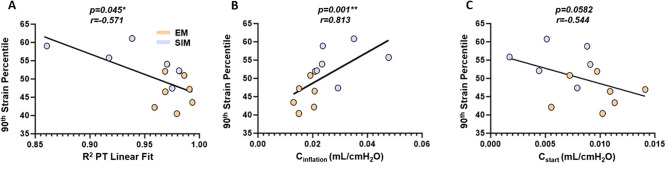
Results of the Spearman’s correlation test between 90th percentile strain and global metrics: (**A**) R^2^ linear PT fit, (**B**) inflation compliance and (**C**) starting compliance, where r represents the strength of correlation [41]

Correlation tests between the 90th percentile strain value and global metrics revealed a significant, moderate, negative correlation with the R^2^ PT linear fit value, a highly significant, strong, positive correlation with inflation compliance, and trends a moderate, negative correlation with starting compliance (Fig. 9A-C).

## Discussion

In this study, we utilize associations between lung global pressure-volume curves to regional local strains via a novel technique to contrast the continuous mechanics of two recruitment maneuvers for the first time. We find significant organ-level differences between EM and SIM in starting compliance, inflation compliance and the degree of linearity of the pressure-time curve. These findings correspond to disparities observed in the evolutionary progression of localized strain distribution throughout inflation and significant differences between the recruitment maneuvers in the 75th percentile strain, 90th percentile strain, and the strain range.

### Inflation: homogenous versus heterogeneous recruitment

Lung surface strain heterogeneity may be linked to sites of damage through increased atelectrauma, as prior work with synchrotron imaging has associated the presence of atelectasis with heterogeneous inflation [42]. Although this is considered a potential consequence of recruitment maneuvers, regional surface strain distribution during inflation for these maneuvers has not been studied previously [43]. We observe heterogenous inflation with SIM, with a greater fraction of low surface strains which progress to populate higher strains at maximum inflation, in comparison to the more unimodal strain distribution of EM (Fig. 4C and D). Unifying the local heterogeneous SIM recruitment with global observations further substantiates this finding: the global PV response for SIM shows a pronounced two-limb inflation, exhibited through significantly lower starting compliance and significantly higher inflation compliance, in comparison to EM (Figs. 2B and 3); this disparate EM and SIM behavior is notable since the PV curve is theorized to represent the recruitment of the underlying alveolar groups, therefore implying only previously opened groups are inflated until SIM’s inflection point, wherein additional groups are then recruited abruptly (31–32, 44).

In contrast, EM’s PV curve demonstrates a more linear response (Fig. 2B). This, in tandem with the significantly higher starting compliance, may suggest a more gradual alveolar recruitment occurs through the stepwise escalation increments, as prior studies have noted an increase in starting compliance for lungs with more homogeneous air distribution [45]. The EM organ level response corresponds with more homogeneous regional strain distribution throughout the entirety of inflation, demonstrated through a normally distributed strain histogram at each incrementally applied volume (Fig. 4C and E). Furthermore, this finding may indicate that EM more efficiently inflates underlying alveolar groups, including previously closed distal groups.

PEEP increments are often clinically used to implement EM whereas in this study increasing tidal volumes were employed to facilitate analogous inflation stage and loading history comparison between the two maneuvers. Intriguingly, despite this difference, our results correspond with the findings of Cereda et al., where MRI images of PEEP increments resulted in homogenous air distribution and inflation of distal alveolar groups [15]. This agreement may be explained by prior work comparing tidal volume increments with PEEP increments, which only demonstrated marginal differences [46]. Furthermore, our findings of more heterogeneity with SIM provides justification and a mechanistic explanation for prior work by Felix et al., which found that abrupt inflation was coupled with more alveolar damage and greater heterogeneity, in contrast to the reduced damage from incremental increases in tidal volume over a short adaptation time [23].

### Pressure-time curve linearity and temporal dependencies

Lung recruitment is dependent on pressure and time, as the viscoelastic property of the lung implies time is required to distribute and disperse increasing air pressures [47–51]; therefore, if inflation to a high peak pressure occurs abruptly, air will disproportionately flow to previously opened alveolar groups, causing overdistension adjacent to closed alveolar groups and higher shear forces, linked to atelectrauma [15, 52]. As a result of these known effects, previous clinical research quantifies the role of pressure and time on lung recruitment via the PT curve, and has linked curve linearity (R^2^), associated with the clinical metric of a stress index value of one, with homogenous alveolar ventilation, improved oxygenation, decreased overdistention and safer recruitment (34–35).

Such global PT concepts can be taken one step further by comparing the disparate linearity of the PT response between EM and SIM recruitment mechanics and the associated diverging localized lung mechanics: EM exhibits significantly better R^2^ linear fit to the PT curve, corresponding to the observed homogeneous local strain response. In contrast, the PT curve of SIM demonstrates an increased downward concavity, aligning with the exhibited heterogeneous regional strains, and supporting the theory that such a concavity indicates the occurrence of non-homogenous tidal recruitment, higher shear forces, and atelectrauma [35, 53]. Our advanced quantification technique of localized measurements of pulmonary surface strains corresponding to the disparate EM and SIM mechanics helps to substantiate previously theorized notions regarding the global temporal pressure response and stress index.

### Strain distribution and stress raisers

The clinical literature predicts the occurrence of pressure inhomogeneities due to the abrupt recruitment of alveolar groups — referred to as mechanical stress raisers or pressure multipliers — causing areas of higher localized stress and strain on alveoli [54–56]. Our global-to-local characterizations align with these predictions, where SIM exhibits a drastic pressure-volume inflection associated with alveoli recruitment (e.g., significantly lower starting compliance followed by higher inflation compliance compared to EM), and where we quantify more of the lung surface experiences high strain values as a result (e.g. greater populations of SIM strain values exceed 40%, along with significantly greater strain ranges compared to EM; Fig. 5). These regions of high strain correspond to localized inhomogeneous areas of high stress and pressure, which have been conjectured to cause injury and be associated with VILI more so than the existence of high global pressures alone [54, 57–60].This study offers clinical insights into lung mechanics for abrupt recruitment or changes to lung ventilation versus the application of high global pressures, as we affirm heterogeneity and overdistension are associated with abrupt inflation in comparison to gradual inflation despite matched applied volumes and global peak pressures.

### Strain anisotropy from recruitment maneuvers

Strain anisotropy has been shown to be a potential disease marker in humans, suggesting evaluating tissue distortion may offer insights regarding the damage incurred by one recruitment method over another [25, 38]. In this study, both SIM and EM maneuvers were applied to healthy lungs, not diseased states, and yielded nearly identical ratios; this suggests that while SIM may have non-uniform strain distributions compared to EM, the average response of each point on the lung surface does not necessarily see a more drastic discrepancy between the two principle stretch magnitudes depending on the recruitment scheme. We note that the strains measured demonstrate anisotropy (measuring < 1) and align with a prior study by Mitzner et al., which found that healthy mouse lung parenchyma has an anisotropic nature [61].

### Unification of global to local recruitment mechanics

The continuous EM and SIM local mean strain values associated with lung pressure follow the same trajectory as the global pressure-volume curve; these results agree with past studies using the same technique, as the lung mean strain has recently been shown to correlate with lung volume [17]. These local strain measures versus the global pressures demonstrate a pronounced two-limb SIM curve which is rather convex, whereas EM demonstrates a more linear response (Fig. 8). Evaluating the continuous 75th and 90th percentile strain values versus the global pressures isolates the regions of high strain to investigate the potential for regional overdistension progression; and we observe that, notably, the 75th percentile and 90th percentile plots indicate increased tangents but similar trends to the mean strain versus pressure graph; this suggests that rapid inflation and higher slopes on the PV curve correlate to steeper increases in regional high strain values (Fig. 8C and D), and align with prior theories from global recruitment studies that previously opened proximal alveoli undergo expansive strain much quicker than alveoli only commencing engagement [31, 44]. This local-to-global analysis finds EM percentile plots versus the pressure reveal the slope decreases at approximately 20 cmH_2_O and appears rather concave, potentially as more of the distal alveoli inflate and reduce the peak strains, whereas in SIM, the slope increases in value as the pressure rises, indicating increasing overdistension in localized areas and potential sites for VILI occurrence and progression [43].

### Limitations

The work divulges continuous regional deformation data via DIC as associated with the global pressure-volume mechanics of recruitment maneuvers, however, such high spatiotemporal image collection necessitates a tradeoff where the continuous and regional strain patterns can only be measured ex-vivo, and therefore without the presence of a chest cavity or circulation. This limitation is partially mitigated by using mice specimens, since murine chest cavities are known to be rather compliant [25]; while the absolute deformation values observed with a chest cavity may be altered, the relative regional patterns and strains behaviors are anticipated to be similar based on in-vivo and ex-vivo comparisons (25, 60, 62–63). Additionally, the utilized breathing frequency of 20BPM is slower than mouse respiratory rates [25, 64], but more physiological than prior similar quasi-static studies and enables DIC measurements, as well as neglects air flow resistance, to allow the assessment of the elastic properties of the lung from pressure-volume curves (65–66). While mouse specimens are used to enable comparisons to prior studies, extrapolations of these findings to the clinic are not yet warranted as recent substantial ventilation differences have been noted between animal models and transplant-rejected human lungs [67–69].

## Conclusions

By inaugurating the continuous quantification and association of local-to-global EM and SIM mechanics, we find evidence of greater regional strains and inhomogeneity linked to overdistension and stress raisers in SIM, indicating the potential for greater damage comparatively incurred by SIM versus EM (10–11). While SIM may no longer be recommended in practice, quantifying its comparative performance here reveals the deleterious impact of its application, as well as substantiating the theories supporting the clinical use of EM. Despite requiring extended application times, the more gradual EM process accounts for the prior ventilation pattern [70] and supports adjustment during application by managing the escalation pressure and volume. Future works aim to optimize the application of recruitment maneuvers by leveraging these experimental characterizations to develop improved finite element breathing models and predictive tools to enable safer maneuvers for differing stages of disease progression and illness, such as to mitigate heterogeneous inflation and damage from ARDS conditions [71, 72].

## Data Availability

No datasets were generated or analysed during the current study.

## Abbreviations

EM: Stepwise escalation maneuver
SIM: Sustained inflation maneuver
VILI: Ventilator-induced lung injury
DIC: Digital image correlation
ARDS: Acute respiratory distress syndrome
BPM: Breaths per minute
MV: Mechanical ventilation
PEEP: Positive-end expiratory pressure
PV: Pressure-volume
